# Quantification of Hepatic Lipid Using 7.0T Proton Magnetic Resonance Spectroscopy and Computed Tomography in Mild Alcoholic Steatotic Mice

**DOI:** 10.4172/2167-0889.1000234

**Published:** 2018-12-31

**Authors:** Qi Cao, Su Xu, Shujing Li, Minjie Chen, Xicui Sun, Yamin Wan, Liya Pi, Zhekang Ying, Bin Ren

**Affiliations:** 1 Department of Diagnostic Radiology and Nuclear Medicine, University of Maryland School of Medicine, Baltimore, Maryland, USA; 2 Department of Radiology, The first affiliated Hospital of Hebei Medical University, Shijiazhuang, Hebei province, PR China; 3 Department of Radiology, the First Affiliated Hospital of Zhengzhou University, Zhengzhou, Henan province, PR China; 4 Department of Pediatrics in the College of Medicine, University of Florida, Gainesville, Florida, USA; 5 Department of Medicine, University of Maryland School of Medicine, Baltimore, Maryland, USA; 6 Department of Surgery, University of Alabama at Birmingham School of Medicine, Alabama, USA

**Keywords:** Alcoholic hepatic steatosis, Proton magnetic resonance spectroscopy, Computed tomography, Liver density, Percentage of liver water, Triglyceride, Cholesterol

## Abstract

**Background:**

In vivo proton magnetic resonance spectroscopy (^1^H MRS) has been used to semi-quantify hepatic lipids in preclinical and clinical studies of fatty liver disease. Quantifying absolute amount of liver lipids utilizing ^1^H MRS and computerized tomography (CT) is essential to accurately interpret hepatic steatosis.

**Purpose:**

To establish reliable parameters to convert relative hepatic lipid levels obtained by 1H-MRS and liver volumes by CT to the absolute amount of liver lipids in a mild hepatic steatosis, and to determinate the correlation between these absolute liver lipids with liver triglyceride (TG) and cholesterol (Chol) measured by biochemistry assays.

**Methods:**

Mild steatosis was induced in mice by a 3 week ethanol diet containing standard lipids. Evaporated liver water was measured after baking liver tissues and volume of liver was measured using water displacement. 1H MRS semiquantitation of hepatic lipids and CT measurement of liver volume were performed and then used to calculate amount of liver lipids. These data were compared with liver TG and Chol.

**Results:**

Percentage of liver water and liver density were persistent in two groups and were used to convert the percentage of liver lipids to liver water by 1H-MRS to the absolute amount of liver lipids per gram of liver or per milliliter of CT volume. Using 1H-MRS and biochemical assays, an increase of liver lipids was confirmed in mild steatosis mice compared to controls (P<0.01). The amounts of imaging detected liver lipids were strongly correlated to liver TG and Chol measured by biochemical assays in mild steatosis mice.

**Conclusion:**

1H MRS and CT liver imaging techniques are able to quantify absolute hepatic lipid levels utilizing relative persistent parameters percentage of liver water and liver density in a preclinical mild steatosis setting.

## Introduction

In the United State and Europe, nearly 10% of the general population is either an excessive amount of alcohol or alcohol dependent [1]. In up to 20% of alcoholics and heavy drinkers, chronic alcohol consumption can lead to alcoholic liver disease (ALD), which refers to a spectrum of alcohol-related liver injuries including hepatic steatosis and fatty liver [2]. Accurate determination of liver lipid content using an *in vivo* quantitative measurement method is essential for managing steatosis in the early stage before liver damage progresses to more severe alcoholic hepatitis and reversible fibrosis, or worse, irreversible alcoholic cirrhosis or cancer.

Many noninvasive imaging techniques including ultrasound, computed tomography (CT), magnetic resonance imaging (MRI), and ^1^H magnetic resonance imaging (^1^H MRS) offer several advantages over biopsy, which is currently the gold standard in the detection and quantification of ALD for the diagnosis and staging of fatty liver disease [3–6]. Conventional ultrasound is widely used as a semi-quantitative assessment tool of lipids in liver tissue, but it is limited by operator dependency and low sensitivity and specificity [7]. CT is one of the most popular modalities clinicians use to make a diagnosis of alcoholic steatosis, but it is associated with significant radiation exposure which limits its use. A study by Park et al. reported the diagnostic performance of unenhanced CT for quantitative assessment of macrovesicular steatosis was not clinically acceptable [8]. MRI provides a sensitive and semi-quantitative assessment of fat tissue, but it cannot present a precise and absolute metabolite quantification of intrahepatic fat [9]. All these modalities have limited capabilities to measure mild steatosis.

*In vivo* ^1^H MRS is the most accurate method to measure fatty infiltration in the liver and its safety has been demonstrated. It has been used as the reference standard for the relative percentage or ratio of hepatic lipid levels to total liver water in various models of steatosis and in patients with alcoholic steatosis and non-alcoholic steatosis [10, 11]. However, the ability to measure the absolute amount of total liver lipids (moles of lipids per gram of liver tissue or moles of lipids per milliliter of liver tissue) would be desirable for clinicians to accurately interpret hepatic steatosis, especially in light of the movement toward precision and personalized clinical medicine. In a prospective study of patients with an increased risk for developing type 2 diabetes mellitus, the liver volume was determined at the beginning and after 6 months of a calorie restricted diet by three dimensional MRI, and intrahepatic lipids were quantified by volume-selective MRS in single voxel stimulated echo acquisition mode (STEAM) [12]. Water displacement based on the principle of Archimedes is the gold standard for volume determination, including liver volume measurement.

However, the organ has to be removed and the volume measured *ex vivo* and in an unperfused state, which limits its application. As a noninvasive imaging technique, CT volumetric measurement using CT datasets has been used to measure the volume of the liver *in vivo* and has demonstrated a strong correlation between the CT volume of the liver and water displacement [13]. Based on this, in this study we measure the liver lipid per liver unit volume using CT imaging. The aims of this study specifically designed in a preclinical model of mild alcoholic steatosis are to convert percentages of lipid to water in liver tissues obtained by^1^H-MRS to absolute amounts of liver lipids (moles/gram liver), with *ex vivo* measurement of total liver water, wet liver weight and liver volume; to calculate absolute amounts of liver lipids (moles/milliliter of CT liver volume) from converted data of ^1^H-MRS percentages of lipid to water in liver tissues and CT liver volume; and to correlate absolute liver lipids with liver triglyceride (TG), cholesterol (Chol), and TG + Chol measured by biochemistry assays.

## Materials and Methods

### Animals and mild alcoholic hepatic steatosis model

Female BALB/C mice (18–20 g, 6 weeks of age) were purchased from Charles River Laboratories (Wilmington, Massachusetts). The mice were housed in a sterile plastic cage under controlled conditions (temperature, 20–22°C and humidity, 50 ± 10%). The experiment was approved by the Institutional Animal Care and Use Committee of the University Of Maryland School Of Medicine and performed in accordance with the National Institutes of Health and our university’s Guide for the Care and Use of Laboratory Animals. Mild liver steatosis was induced in mice fed with ethanol liquid diet containing fixed percentages of fats for 3 weeks according to the modified method of Xu et al [14]. Fat composition of the liquid alcohol diet is linoleic (C18:2) 9.3 g/L, linolenic (C18:3) 0.3 g/L, total saturated fat 5.2 g/L, total monounsaturated fat 23.5 g/L and total polyunsaturated fat 9.7 g/L. Proximate profile of liquid alcohol diet is fat 30.5% and 359 Kcal/L.

Hepatic steatosis was assessed by the percentage of hepatocytes with visible steatosis; mild steatosis was defined as 5–33% of hepatocytes affected [15]. Eight female mice were randomly divided into two groups. The mice were sacrificed 15 hours after liver imaging, and blood samples were immediately withdrawn from the left ventricle. Plasma triglyceride (TG, Biovision, Milpitas, CA) and cholesterol (Chol, Biovision, Milpitas, CA), alanine aminotransferase (ALT, Sigma-Aldrich, St Louis, MO), and aspartate aminotransferase (AST, Sigma-Aldrich, St Louis, MO) levels were determined according to the methods of Cao et al. [16]. Liver tissues were removed for measurement of liver volume by water displacement [13], liver water by baking spliced liver samples in a 60°C oven for three days, histological examination by H&E staining, and lipids by biochemistry assays.

### T ^1^H-MRS measurements of liver lipids and data analyses

^1^H-MRS semi-quantification of liver lipids were performed on a Bruker BioSpec 70/30USR Avance III 7 T horizontal bore MR scanner (Bruker Biospin MRI GmbH, Ettlingen, Germany). During the experiment, all mice were anesthetized with isoflurane/air at 1 to 2%/L/min oxygen) with respiratory monitoring. MR images were collected using multi-slice proton-density-weighted and T2-weighted rapid acquisition with relaxation enhancement sequence with TR=3000 ms, TE1/TE2=9.2/27.6 ms, slice thickness=1mm, number of slices=12, field of view=3.5 × 3.5 cm^2^, matrix size=128 × 128, number of excitation (NEX) = 2. The volume of interest of the subsequent prone spectroscopy was carefully located on homogeneous liver parenchyma to avoid contributions from obvious blood vessels, subcutaneous fat, and air. The single-voxel was performed using a point-resolved spectroscopy sequence without water suppression with the following parameters: voxel volume 4 × 6 × 4 mm^3^, TR=10000 ms, TE=16.5 ms, 64 signal averages. All MR spectra were processed with Bruker Topspin package. In the mouse model, the distinguished lipid peak was the Lip13 resonance (originating from the (CH2) protons at 1.3 ppm) which represented the major resonance and accounts for approximately 70% of the total lipid signal for fatty liver (17). For this reason, we used it to quantify the fat content of our animal model. The fat fraction (FF) was calculated from the MR spectra as the ratio of the Lip13 resonance area (Lip13) relative to the water peak area.

### CT imaging and water displacement liver volumetric measurement

For CT liver volume measurement, CT scans were performed on an Inveon micro-PET/CT scanner (Siemens Medical Solutions USA, Inc.) equipped with 38 mm width bed for mice whole-body imaging at the MicroPET/CT Imaging Laboratory at the Core for Translation Research Imaging @ Maryland (C-TRIM). The hepatic volumetric measurement was created by CT using the summation-of-area method on Simens Inveon Research Workplace [13]. For ex vivo measurement of liver volume, after the liver of each mouse was weighed, the whole liver tissue was put into a cylinder full of water and the liver volume (milliliter) was obtained according to the volume difference between the water height scales before and after liver placement. Then the liver volume by either CT or displacement water was used to calculate liver density (grams per milliliter).

### Calculation of absolute liver lipids with data from *ex vivo* measurement of liver water and liver volume

About 500 milligrams (mg) of liver tissue from each animal liver was cut and placed in a 60°C oven for 3 days. Dry liver weight was measured at the end of each day and the amount of water evaporation from the liver tissue was calculated by deduction of dry liver weights from wet liver tissue weights and percentage of water to liver was calculated. Then the total liver water of each animal was calculated by multiplying the whole liver tissue weight of each animal with the percentages of water in its spliced liver tissue. Total water in grams was converted into moles in the whole liver by multiplying total water in gram by 0.0555 (1 gram of water equals 0.0555 moles). Total liver lipids in each animal were calculated by multiplying moles of total liver water of each animal with the percentages of lipid to water in liver tissues which were acquired by liver ^1^H-MRS. The unit of liver lipid was expressed as moles of lipid per gram of liver tissue or per milliliter liver tissue.

### Statistics

All the values are represented as the mean ± standard error of the mean (SEM). Student’s t-test was used to determine differences between the two groups.

Correlation analysis was performed between data of total liver lipids by MRS and the data of liver or blood lipids by biochemical assays. The same method was used to analyze MRS-based data of total liver lipids per milliliter of liver and the data of liver or blood lipids by biochemical assays between the two groups. In addition, data of liver volumes by CT and by water displacement underwent correlation analysis. All analyses described here were conducted using Excel software. P < 0.05 was considered statistically significant.

## Results

### *In vivo* liver ^1^H-MRS in a mild alcoholic hepatic steatosis

**Figure 1:**
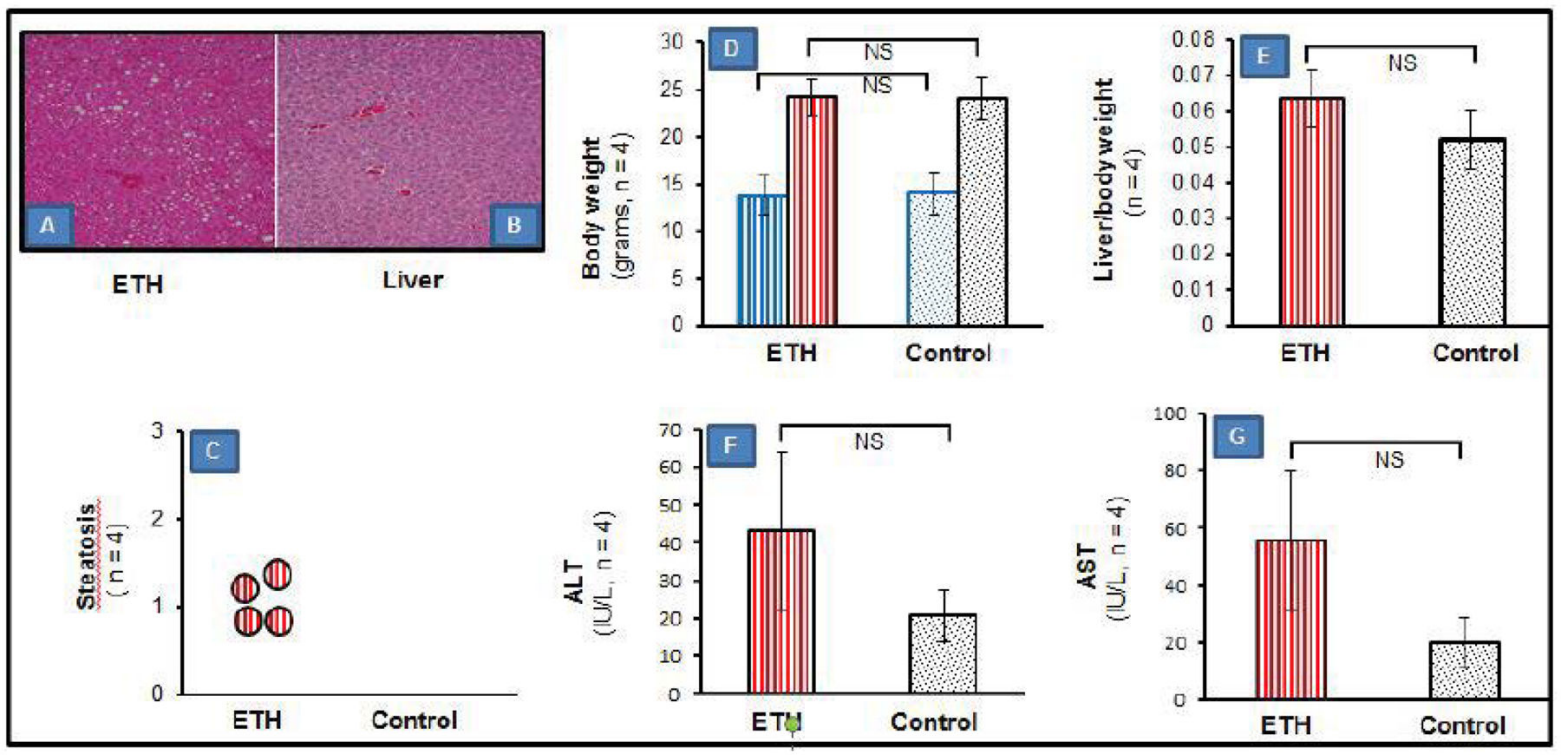
Hepatic histopathology and plasma biochemical changes. (A,B) H & E stained liver sections in ETH-fed (A) and control (B) mice (n=4). Magnification is 100×. (C) Visible increase in steatosis in ETH-fed mice compared to the control mice with no visible fat deposits. (D) Body weight changes at the beginning of ETH feeding and the end of ETH feeding. (E) Ratio of liver to body weight at wk 3 after ETH feeding in the ETH-fed and control mice. (F, G) ALT and AST levels in the ETH-fed and control mice. Data presented as mean ± standard error of the mean. (NS, no significance; ALT, alanine aminotransferase; AST, aspartate aminotransferase; ETH, ethanol diet-fed mouse).

H&E liver staining showed fat accumulation corresponding to a mild clinical steatosis score in ethanol-fed mice (less than 20%). Control mice had no visible hepatic fat deposits (Figure 1A–1C). Body weight gain and the ratio of liver to body weight were not different between the mild steatosis and control mice (Figure 1D and 1E). ALT and AST levels released from damaged hepatocytes tended to increase in mice with mild steatosis but no statistical difference was found when compared to controls (Figure 1F and 1G).

**Figure 2:**
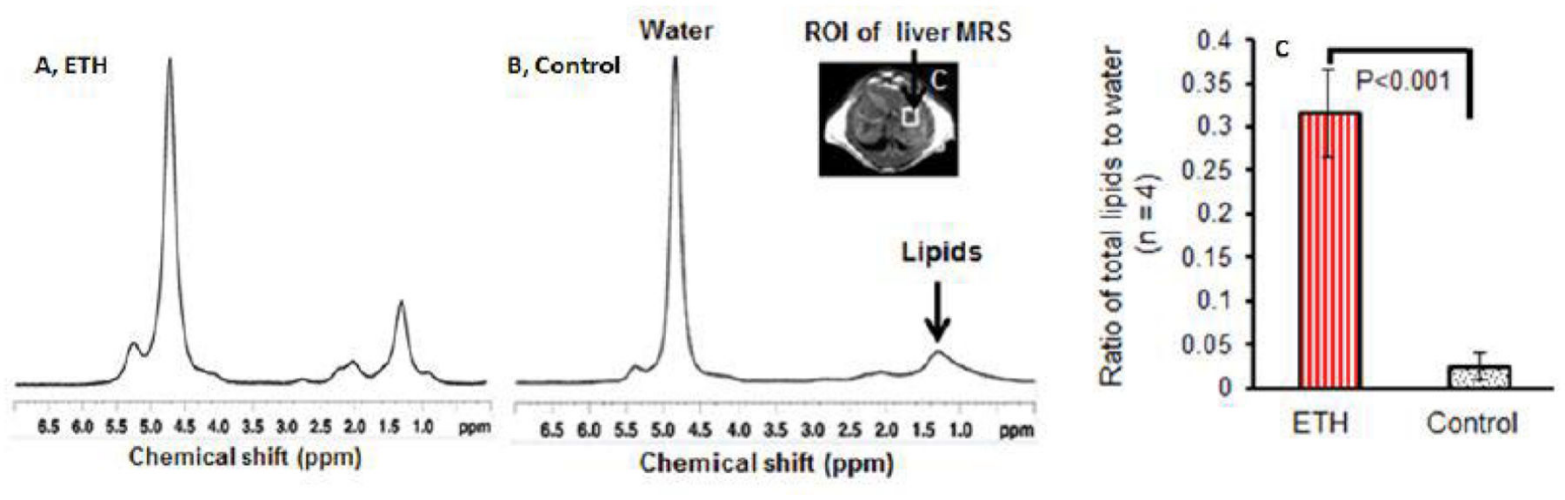
Representative *in vivo* ^1^H-MR spectra and corresponding voxel locations. (A) Liver water and lipid profile from a 3-wk ETH-fed BALB/C mouse. (B) Liver water and lipid profile from a control mouse. (C) ROI of liver MRI acquiring location. (D) Comparison of the ratio of liver total lipids to liver water by MRS between ETH-fed and control mice. The water peak heights were used to normalize the MR spectra as there was no statistical difference in the water peak heights between the groups in the measured regions. Data presented as mean ± standard error of the mean. (wk, week; ROI, region of interest; MRS, magnetic resonance spectroscopy; ppm, parts per million; ETH, ethanol diet-fed mouse).

Accurate lipids peak located at 1.3 ppm and water peak located at 4.7 ppm were acquired both in disease model and controls. There was no statistical difference in the water peak levels between the two groups in the regions measured (Figure 2A and 2B). The percentage of the liver lipids to total liver water was calculated. The percentages of liver lipids to total liver water was higher in the disease model mice than in controls (0.32 ± 0.07 versus 0.03 ± 0.005, p<0.001) (Figure 2C).

Absolute amount of total liver lipids calculated from *ex vivo* measurement of liver water and percentages of liver lipid to total liver water by *in vivo* ^1^H-MRS, and plasma and liver TG, Chol, and TG + Chol levels by biochemistry assays in mice with mild alcoholic steatosis

**Figure 3:**
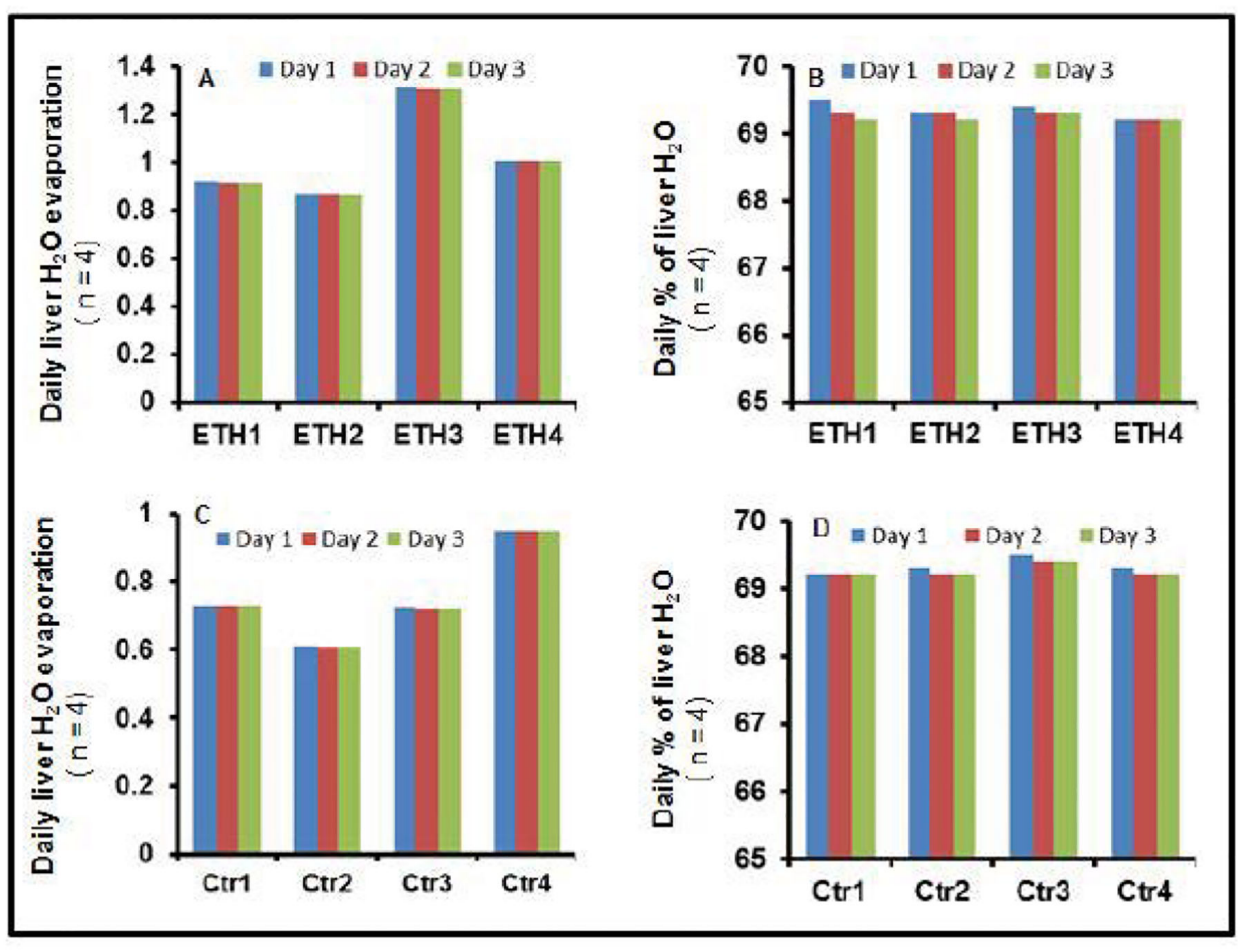
Representative calculations of daily liver water evaporation and daily percentages of liver water to wet liver weight. (A) Representative daily liver water evaporation in grams of 4 ETH-fed BALB/C mice (ETH1–4) at day 3 water evaporation in a 60oC oven. (B) Representative daily percentages of liver water of 4 ETH-fed BALB/C mice (ETH1–4). (C) Representative daily liver water evaporation in grams of 4 control BALB/C mice (Ctr1–4) at 3-day water evaporation in a 60°C oven. (D) Representative daily percentages of liver water of 4 control BALB/C mice (Ctr1–4). (ETH, ethanol diet fed mouse; Ctr, control mouse).

Figure 3 shows the *ex vivo* measurement of liver water (A,D), % of liver water (B,E) and liver lipids (C,F) in the two groups at day three of baking. Our data showed no significant difference in absolute water weight (moles) in each individual sample at the end of each day. This indicates that almost all water evaporated from the liver after one day of oven baking. Percentages of liver water at the end of each day were relatively persistent between the two groups. The total liver lipids in moles showed a similar pattern in individual mice in day 3 of oven baking.

**Figure 4:**
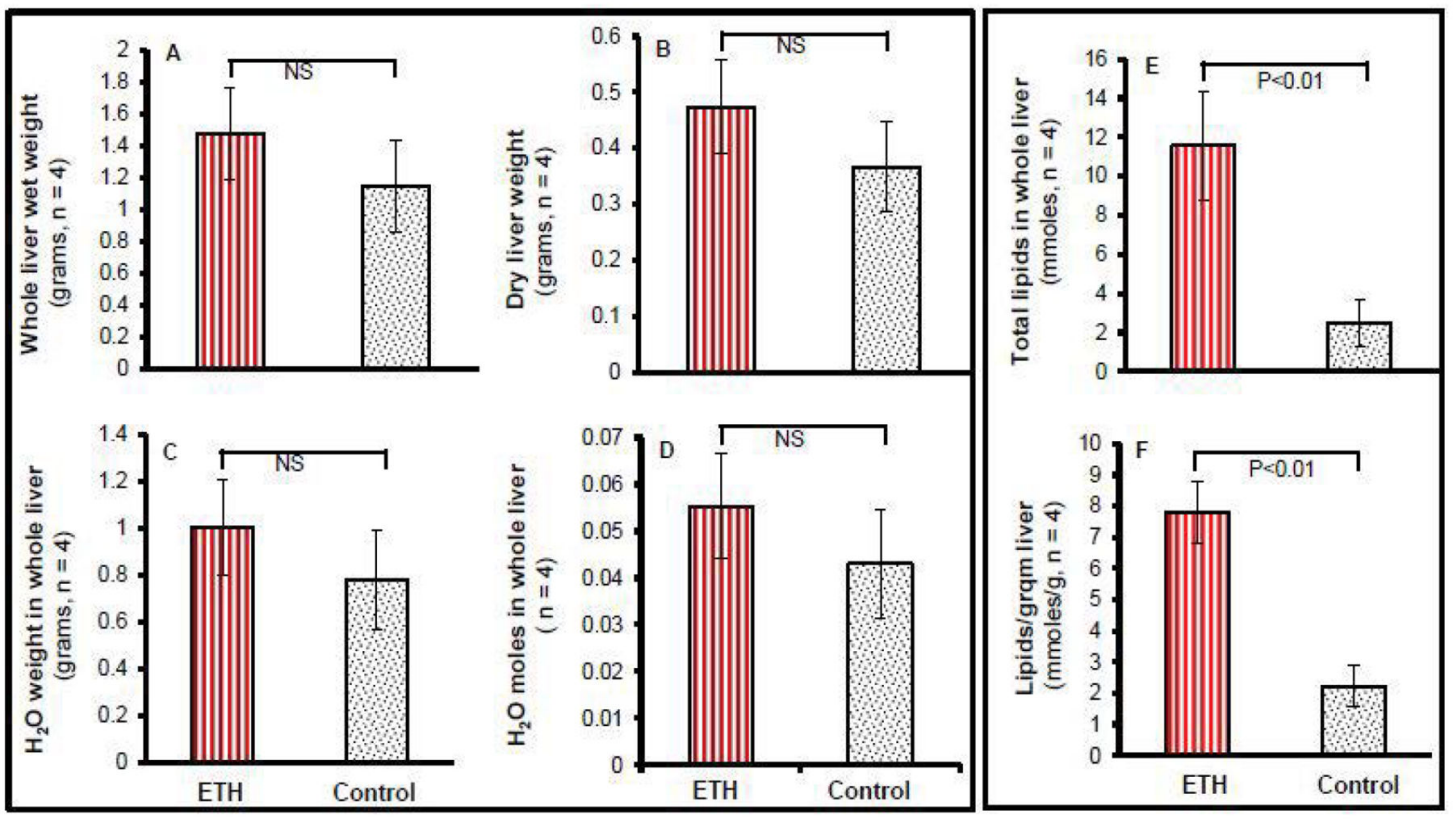
Accurate amounts of liver lipids calculated by total liver water weight and percentages of lipids-to-water on MRS. (A) Total liver wet weight in grams in 3-wk ETH diet-fed BALB/C and control mice. (B) Dry liver weight in grams in 3-wk ETH diet-fed BALB/C and control mice after 3 day water evaporation in a 60°C oven. (C) Water weight in the whole liver in grams after deducting dry liver weight from the whole liver wet weight. (D) Total water (moles) in the whole liver from the same mice after converting grams of water weight in the whole liver to moles of water in the whole liver. (E) Total liver lipids (mmoles) in the whole liver from the same mice after multiplying total water (mmoles) in the whole by ratio of liver lipids to water. (F) Liver lipids (mmoles) per gram of liver from the same mice after dividing total lipids (mmoles) in the whole liver by the hepatic wet weight. Data presented as mean ± standard error of the mean.

In our summarized data from the two groups at day 3, there was no significant difference in the amounts of weight of whole wet liver tissue (1.45 ± 0.33 versus 1.17 ± 0.37 grams) or dry liver tissue (0.47 ± 0.12 versus 0.36 ± 0.14 grams) (Figure 4A and 4B), nor in total liver water in grams or in moles (Figure 4C and 4D). However, compared to their controls, ethanol-fed mice showed a significant increase in total liver lipids (11.8 ± 2.1 mmoles and 2.3 ± 0.7 mmoles, P<0.01, Figure 4E) and liver lipids per gram of liver tissue (7.8 ± 0.9 versus 2.1 ± 0.6 mmoles/g, P<0.01, Figure 4F).

**Figure 5:**
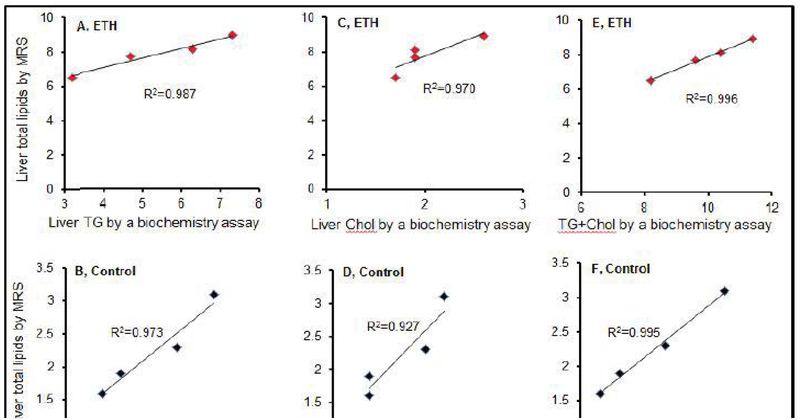
Levels of triglyceride and cholesterol in plasma and liver by biochemistry assays. (A, D) Liver and plasma triglyceride levels in 3-wk ETH liquid diet-fed BALB/C and control mice. (B,E) Liver and plasma cholesterol levels in 3-wk ETH liquid diet-fed BALB/C and control mice. (C,F) Combination of triglyceride and cholesterol levels in 3-wk ETH liquid diet-fed BALB/C and control mice. Data presented as mean ± standard error of the mean. (TG, triglyceride; Chol; cholestrol; mmoles, micromoles).

Ethanol-fed mice had a significant increase in levels of liver TG (5.2 ± 1.6 versus 1.2 ± 0.2 μmoles/g, P<0.001, Figure 5A), liver Chol (2.1 ± 0.5 versus 0.8 ± 0.2 μmoles/g, P<0.001, Figure 5B), and liver TG + Chol (P<0.001, Figure 5C). Although not for plasma Chol (Figure 5E), there was significant difference in plasma TG (2.4 ± 0.6 versus 0.8 ± 0.1 μmoles/mL, P<0.01, Figure 5D) and plasma TG + Chol (P<0.001, Figure 5F) between the two groups.

### Correlation between total liver lipids converted from ^1^H MRS data and levels of TG, Chol, and TG + Chol in liver

**Figure 6:**
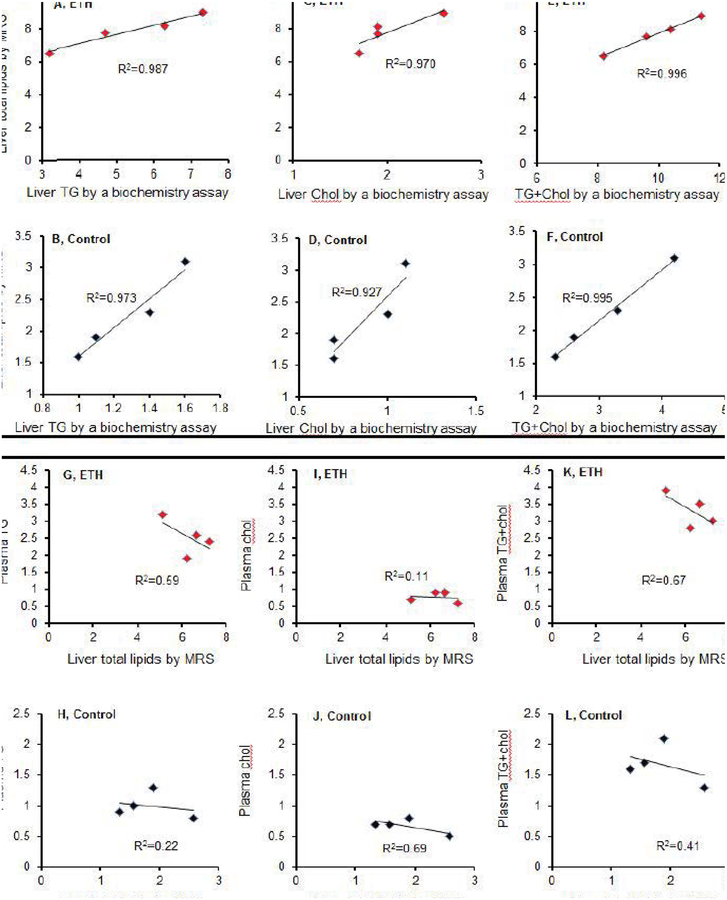
Correlation of total liver lipids by MRS with triglyceride, cholesterol, and TG+Chol by biochemistry assays. (A,G) Correlation between total liver lipids by MRS and liver and plasma triglyceride levels in 3-wk ETH liquid diet-fed BALB/C mice. (B,H) Correlation between total liver lipids by MRS and plasma and plasma triglyceride levels in control mice. (C,I) Correlation between total liver lipids by MRS and plasma and liver cholesterol in 3-wk ETH liquid diet-fed BALB/C mice. (D,J) show correlation between total liver lipids by MRS and liver and plasma cholesterol in control mice. (E,K) Correlation between total liver lipids by MRS and combination of liver and plasma triglyceride and cholesterol levels in 3-wk ETH liquid diet-fed BALB/C mice. (F,L) Correlation between total liver lipids by MRS and combination of liver and plasma triglyceride and cholesterol in control mice. Dot presented as number of individual mouse.

There was strong correlation between total liver lipids by ^1^H MRS and liver TG, Chol and TG + Chol by biochemistry assay both in mild steatosis mice (R^2^=0.987, 0.970, and 0.996, respectively, Figure 6A, 6C and 6E) and control mice (R^2^=0.973, 0.927, and 0.995, respectively, Figure 6B, 6D and 6F). However, there was a weak or no correlation between total liver lipids by ^1^H MRS and plasma TG, Chol and TG+Chol in mild steatosis mice (R^2^=0.59, 0.11, 0.67, respectively, Figure 6G and 6K) and control mice (R^2^=0.22, 0.69, 0.41, respectively, Figure 6H, 6J and 6L).

### Calculation of absolute amounts of liver lipid to liver volume measured by CT and amounts of liver lipid to liver volume measured by water displacement in mice with mild steatosis

**Figure 7:**
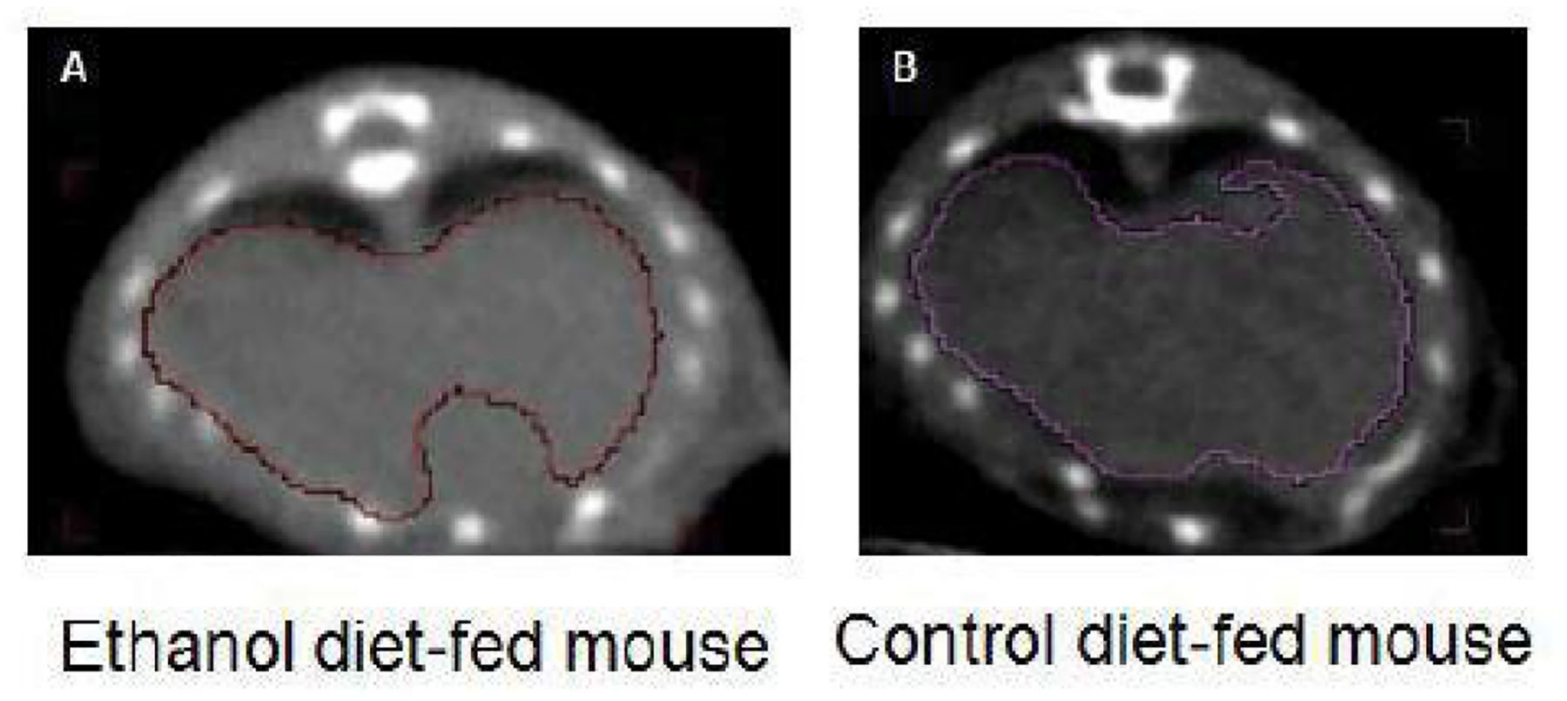
Representative *in vivo* non-contrast CT and corresponding voxel locations. (A) Liver contour used for calculation of liver volumes (mL) from a 3-wk ETH-fed BALB/C mouse. (B) Liver contour used for calculation of liver volumes (mL) from control mouse.

**Figure 8:**
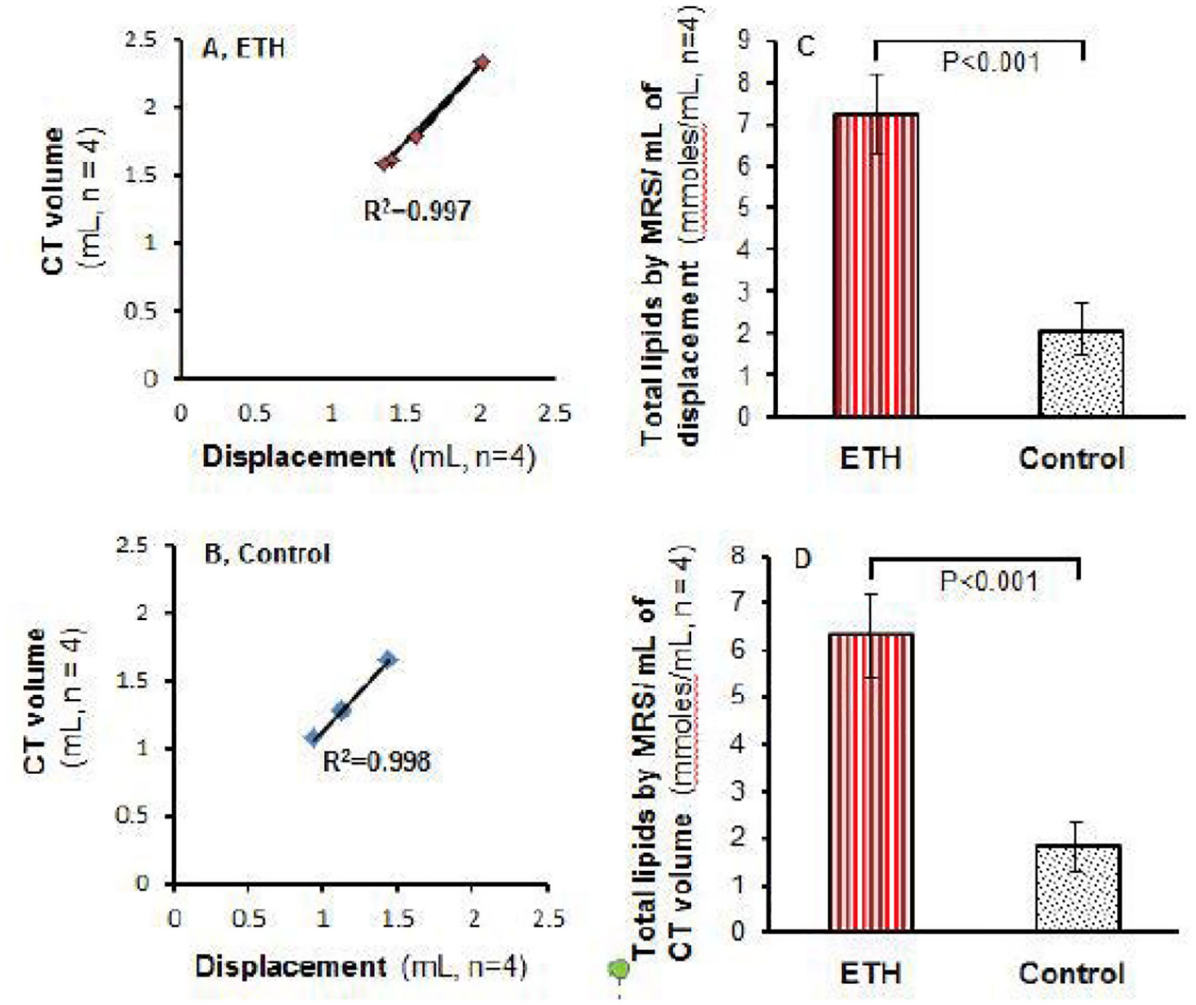
Total liver lipids per milliliter of water displacement and CT volumes of livers of ETH diet-fed and control mice and correlation of displacement and CT volumes. (A) Comparison of displacement volume of livers between 3-wk ETH liquid diet-fed and control mice. (B) Comparison of CT volume of livers between 3-wk ETH liquid diet-fed and control mice. (C) Correlation between CT volumes and displacement volumes in 3-wk ETH liquid diet-fed BALB/C mice. (D) Correlation between CT volumes and displacement volumes in control mice. (E) Difference of total liver lipids per milliliter of displacement volume of livers between 3-wk ETH liquid diet-fed and control mice. (F) Difference of total liver lipids per milliliter of CT volume of livers between 3-wk ETH liquid diet-fed and control mice. Data presented as mean ± SD of the mean of individual mouse in Figs A, B, E and F. (CT, computed tomography; mL, milliliter).

The liver contours used for calculating liver volumes in non-contrast CT and corresponding voxel locations in mild steatosis and control mice are shown in Figure 7. Liver volumes determined by CT were about 14.2 percent higher than the volumes measured by water displacement, but there was no significant difference between the two measurements. No significant difference was found in the density of livers between the two groups of mice representing water displacement (g/ml) and CT volume (g/ml) (Table.1). There was no significant difference in liver water displacement volumes (1.54 ± 0.36 versus 1.16 ± 0.2 ml, P>0.05) and CT volumes (1.78 ± 0.4 versus 1.32 ± 0.24 ml, P>0.05) between the model and control mice, though slight higher liver volume were found by CT measurement than that of water displacement. A strong correlation between CT volumes and the water displacement volumes was found in the disease mice (R^2^=0.997, Figure 8A) and control mice (R^2^=0.998, Figure 8B). In addition, a significant difference in total lipids per milliliter of displacement (7.2 ± 0.8 versus 2.1 ± 0.3 mmoles, P<0.001, Figure 8C) and total lipids by ^1^H MRS per milliliter of CT volume (6.2 ± 0.7 versus 1.8 ± 0.3 mmoles, P<0.001, Figure 8D) was found between the model and control mice.

### Strong correlation between liver lipids per milliliter of liver volume measured by CT and liver TG, Cho and TG+Cho by biochemistry assays

**Figure 9:**
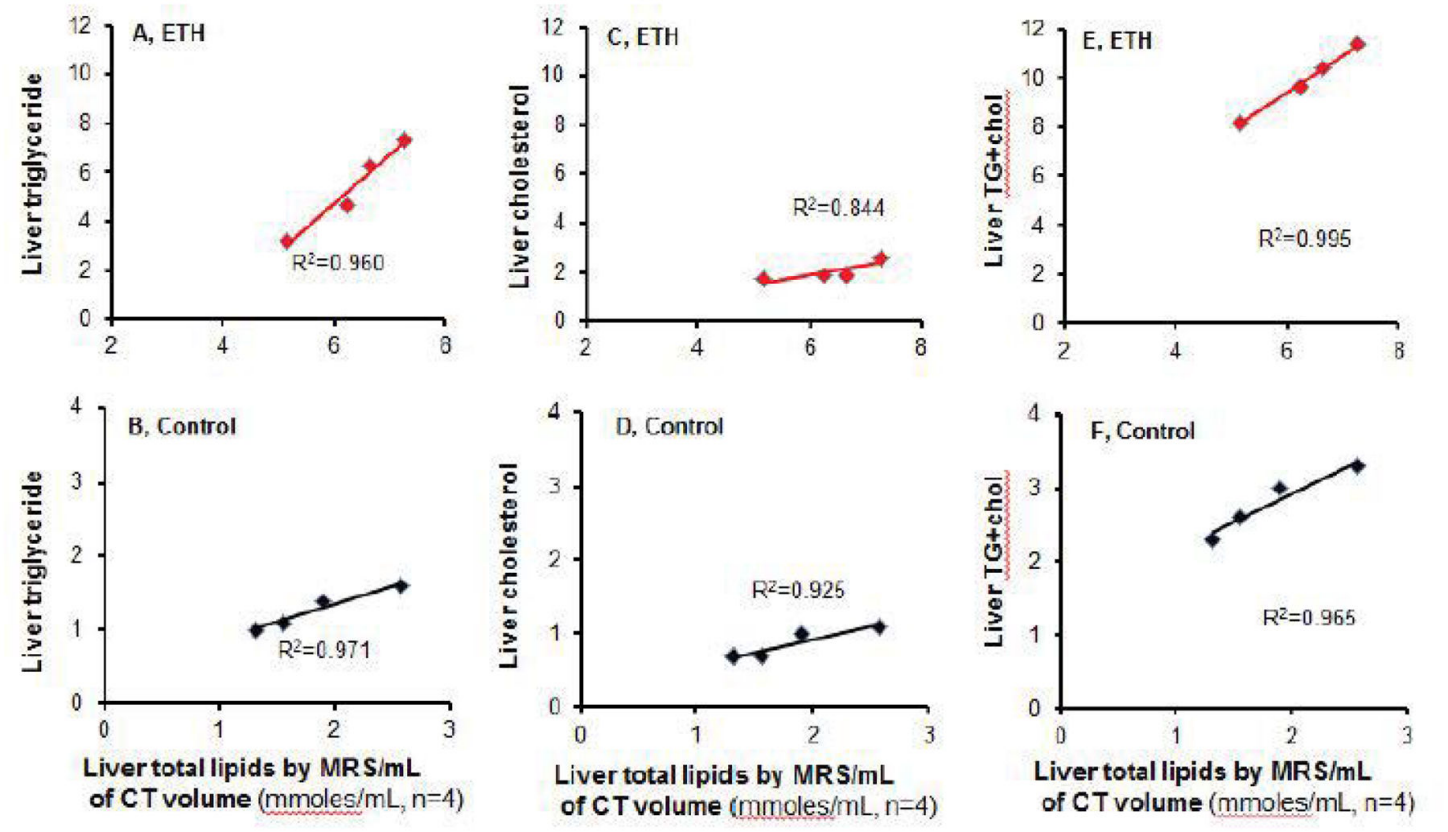
Correlation of total liver lipids by MRS with liver triglyceride, cholesterol, and TG+Chol by biochemistry assays. (A) Correlation between liver total lipids by MRS per milliliter of CT volume and liver triglyceride level in 3-wk ETH liquid diet-fed BALB/C mice. (B) Correlation between liver total lipids by MRS per milliliter of CT volume and triglyceride level in control mice. (C) Correlation between total liver lipids by MRS per milliliter of CT volume and liver cholesterol level in 3-wk ETH liquid diet-fed BALB/C mice. (D) Correlation between total liver lipids by MRS per milliliter of CT volume and liver cholesterol level in control mice. (E) Correlation between total liver lipids by MRS per milliliter of CT volume and combination of liver triglyceride and cholesterol levels in 3-wk ETH liquid diet-fed BALB/C mice. (F) Correlation between total liver lipids by MRS per milliliter of CT volume and combination of liver triglyceride and cholesterol levels in control mice. Dot presented as mean of individual mouse.

After analysis of correlation statistics, a strong correlation between absolute liver lipids by ^1^H MRS and CT volume and liver TG, Chol, TG+Chol was found in the model (R^2^=0.960, 0.844, 0.995, respectively, Figure 9A, 9C and 9E) and control group (R^2^=0.971, 0.925,0.965, respectively, Figure 9B, 9D and 9F). This finding indicates possible replacement of ex vivo liver volume measurement by in vivo CT volume measurement.

## Discussion

In this study we had investigated the accuracy of quantifying absolute amounts of liver lipids by converting ^1^H MRS semi-quantitative liver lipids in a preclinical model of mild alcoholic hepatic steatosis. Using a 7.0 T MR Scanner and CT we demonstrated that ^1^H MRS was capable of discriminating between a clinically relevant degree of mild steatosis and a normal liver in a mild alcoholic steatosis model and its control. We also have shown that the development of mild steatosis in the livers of mice fed with or without ethanol did not significantly change hepatic water weight and ex vivo liver volume. Most interestingly, after ex vivo measurement of liver water and liver volume, we identified two parameters of the percentage of liver water and liver density were persistent in model and control animals and these parameters were useful and might be applied to direct quantify absolute liver lipids. Finally, ^1^H-MRS and CT methods used to calculate hepatic lipids were correlated with measurements of hepatic lipids by biochemical assay, showing this calculation method provides a suitable representation of absolute hepatic lipids in the setting of mild alcoholic hepatic steatosis.

Current clinical non-invasive imaging techniques for the detection of fat content are not capable of measuring mild alcoholic hepatic steatosis and no study of mild alcoholic hepatic steatosis has been validated by biochemical fat determination. Liver biopsy with histology analysis is impractical for detecting mild alcoholic fat liver disease in clinical practice and for monitoring its progression or regression because it is an invasive procedure carrying risks for morbidity and complications.

Ultrasonography has excellent accuracy in assessing moderate to severe steatosis (84.8% sensitive and 93.6% specificity), however, sensitivity decreases dramatically for hepatic steatosis <30 % [3]. Conventional unenhanced CT is also capable of detecting and quantifying moderate to severe steatosis, but it is inaccurate in diagnosing mild liver steatosis and it involves the use of radiation(4). Because it is more effective than ultrasound and CT [5], MRI-based liver proton density fat fraction (PDFF) quantification methods are an important step forward using MRI-based measurement as an accurate biomarker capable of steatosis quantification of the entire liver in animals and humans, including the mild hepatic steatosis population [18,19].

Several studies have shown that in vivo ^1^H MRS is a noninvasive method to detect and semi-quantify a small amount of hepatic fat content in animal models and human livers [6,20]. A study by Mennesson and colleagues showed that 1.5 T ^1^H MRS-obtained hepatic fat-water percentage and steatosis grade were highly correlated(r = 0.852) in patients with nonalcoholic fatty liver disease, alcoholic liver disease, cholangiopathy and autoimmune hepatitis. The sensitivity and specificity of hepatic fat-water ratio to detect moderate fatty infiltration were very high (96% and 93%, respectively); the sensitivity and specificity in populations with mild liver fatty infiltration are not known [21]. To date, no clinical studies have performed validation of ^1^H MRS-obtained hepatic fat-water percentage by biochemistry assay-measured liver lipids. A high linear correlation between ^1^H MRS data and total liver fatty levels with the degree of histopathologic and biochemical steatosis was identified in rats with mild steatosis (<10%). These results indicated that the degree of hepatic steatosis with mild microvesicular steatosis (<10%) could be precisely predicted using 3-T ^1^H MRS [22]. We successfully detected the lipid signal at 1.3 ppm by 7-T ^1^H MRS in mild alcoholic steatosis mice fed with a standard ethanol liquid diet, which is consistent with the findings in rats using methylcholine deficiency and choline deficiency diets by Ou et al. [22]. In our study, we converted percentages of hepatic lipid-to-water into absolute amounts of liver lipids per gram of liver tissue and per milliliter of liver with ex vivo measurement of wet liver weight (g) and liver volume (milliliter). We found liver density calculated by both water displacement and CT volume was relatively persistent in animals with both mild hepatic steatosis and controls. These data suggests this relatively persistent liver density may be a useful parameter that can be used to calculate absolute hepatic lipids in mild hepatic steatosis models. Similarly, the volumes of livers measured by in vivo CT and ex vivo water displacement in two groups were not significantly different, indicating in vivo CT measurement of liver volume may be applicable to represent absolute liver volumes in future studies. A clinical study by Lemke et al. [13] prospectively calculated the expected intraoperative weight and volume of living donors’ right lobes and the volume of intraoperative homologue grafts determined by means of water displacement as the reference standard. They found that all corresponding pre- and intraoperative expected weights in grams and volumes in milliliters correlated significantly with each other [13]. Our data demonstrated the percentage of liver water in terms of liver water weight divided by wet liver weight × 100% was also relatively consistent during a 3 day baking in mild liver steatosis mice and controls. We found in this pilot study of mild liver fat infiltration two parameters of liver density(wet liver weight per mL of liver volume) and the percentage of liver water are relatively persistent. Thus we may use current liver density value with combination of CT measured liver volume to calculate total liver weight, then using the known relative persistent percentage of liver water we can calculate water weight in a similar model and normal mice, finally we can calculate absolute liver lipids by measuring percentage of liver lipid to water with ^1^H-MRS. Thus, our current pilot study opens a new window allowing researchers to establish non-invasive imaging techniques liver ^1^H MRS and liver CT to acutely quantify liver lipids based on our findings of the relative persistence of liver density and percentage of liver water in models of mild alcoholic liver steatosis. Accordingly, we used the ratio of liver lipid to liver water by ^1^H MRS and CT volume in living mice to calculate the absolute amount of hepatic lipids per milliliter of CT volumes of livers, which showed significant correlation with the liver TG, Chol, and TG + Chol by biochemistry assays. These findings suggest the reproducibility of the correlation between the non-invasive imaging analyses to other quantification methods in a mild alcoholic steatosis mice model.

There are some limitations of this technique. The use of the lipid and water signals can be prone to quantification error, caused by different lipid and water contents in liver tissues. This can be potentially problematic due to different T_2_ times. A study by Hamilton et al. has shown that transverse relaxation may create errors in the quantitative analysis of lipid accumulation if water and lipids resonances had different T_2_ relaxation times [23]. The exact peak of different types of hepatic steatosis in the mouse model, including TG and Chol, cannot be separated by 7T MRS [24]. Only intrahepatic lipids are acquired by ^1^H MRS, whereas membrane and extrahepatic lipids are invisible due to their reduced mobility [25]. The sample size in the study is relatively small which may have reduced the statistical power. Further longitudinal studies with a larger in vivo sample size are needed. Lastly, future studies of other animal models and human experiments should be used to validate the results.

We make hypothesis based on our current preliminary data that with the increasing accuracy of higher resolution ^1^H MRS (greater than 9 T MRS), it is likely to become an important clinical tool, potentially becoming the technique of choice for the diagnosis of early-stage alcoholic fatty liver, as well as for clinicians to acutely interpret hepatic steatosis in precision and personalized clinical medicine. Before application this technique to humans, many experiments include liver biopsy of volunteers with mild liver steatosis for assessment of liver lipids and histopathology, and establishment of more detail imaging techniques of liver lipid quantification. Our current methods may provide the mean toward clinical application of quantification of absolute liver lipids.

In conclusion, mild hepatic steatosis can be detected by ^1^H MRS in a mild alcoholic liver steatosis model. In this model, two relative persistent parameters, percentage of liver water and liver density, relative hepatic lipid levels and liver volume with non-invasive liver imaging techniques of ^1^H MRS and CT may be applied to calculate the absolute amount of liver lipids, which may reflect levels of liver lipids of TG and Chol in a preclinical mild steatosis setting.

## Figures and Tables

**Table 1:** Weight, volume and density of liver in 8 mice. 1 to 4 are ETH diet-fed mice, 5–8 are control diet-fed mice.

Mouse #	Liver weight (g)	Displacement (ml)	CT volume (ml)	Liver density		difference (%) of displacement and CT volumes
				g/ml displacement	g/ml CT volume	
ETH 1	1.32	1.41	1.61	0.94	0.82	14.2
ETH 2	1.15	1.16	1.38	0.99	0.83	19.0
ETH 3	1.89	2.02	2.33	0.94	0.81	15.3
ETH 4	1.45	1.57	1.78	0.92	0.81	13.4
		1.54 ± 0.36#	1.78 ± 0.40#			15.5 ± 2.5#
C5	1.05	1.13	1.28	0.93	0.82	13.3
C6	0.88	0.94	1.07	0.94	0.82	13.8
C7	1.04	1.13	1.26	0.92	0.83	11.5
C8	1.37	1.43	1.65	0.96	0.83	15.4
		1.16 ± 0.20	1.31 ± 0.24			13.5 ± 1.6
